# Parenting, Peer Relationships, Academic Self-efficacy, and Academic Achievement: Direct and Mediating Effects

**DOI:** 10.3389/fpsyg.2017.02120

**Published:** 2017-12-15

**Authors:** Anna Llorca, María Cristina Richaud, Elisabeth Malonda

**Affiliations:** ^1^Department of Personality, Evaluation and Psychological Treatment, University of Valencia, Valencia, Spain; ^2^National Council of Scientific and Technological Research, Buenos Aires, Argentina; ^3^Basics Psychology, University of Valencia, Valencia, Spain

**Keywords:** peers relationships, attachment, victimization, academic performance, adolescence, parenting styles, aggression

## Abstract

The aim of the present study is to analyze the relation between authoritative and permissive parenting styles with the kinds of adolescent peer relationships (attachment, victimization, or aggression), and of the latter ones, in turn, with academic self-efficacy, and academic performance, in three waves that range from the early-mid adolescence to late adolescence. Five hundred Spanish adolescents, of both sexes, participated in a three-wave longitudinal study in Valencia, Spain. In the first wave, adolescents were either in the third year of secondary school or the fourth year of secondary school. The mean age in the first wave was 14.70 (*SD* = 0.68; range = 13–16 years). Child Report of Parental Behavior Inventory (Schaefer, 1965; Samper et al., 2006), Peer Attachment (from the Inventory of Parent and Peer Attachment by Armsden and Greenberg, 1987), Victimization (from the Kit at School, Buhs et al., 2010), Physical and Verbal Aggression Scale (Caprara and Pastorelli, 1993; Del Barrio et al., 2001), items of academic self-efficacy, and items of academic performance were administered. Structural equations modeling—path analysis was employed to explore the proposed models. The results indicated that parenting styles relate to the way the adolescents develops attachments to their peers and to academic self-efficacy. The mother's permissive style is an important positive predictor of aggressive behavior and a negative predictor of attachment to their peers. At the end, peer relations and academic self-efficacy are mediator variables between parenting styles and academic performance.

## Introduction

The present article examines through a longitudinal study, whether authoritative and permissive parenting styles are associated with the type of relationships that the adolescent develops with their peers (attachment, victimization, or aggression) and of this in turn with academic self-efficacy and performance. There are several studies that stress the importance of parenting styles (e.g., Chen et al., 1997; Steinberg et al., 2006) and peer relationships (e.g., Iyer et al., 2010) in relation to academic self-efficacy and of the effect of these on academic performance (e.g., Bandura et al., 2001; Schunk and Pajares, 2009). However, there are few studies that consider specifically permissive parenting style, and parenting styles and peer relationships simultaneously in explaining academic self-efficacy and performance and taking into account different moments during adolescence.

## Authoritative and permissive parenting styles, peer relationships, and academic self-efficacy

Parenting styles differ according to the levels of parental sensitivity (i.e., warmth and affection) and parental control (i.e., promoting children's autonomy), and both of these factors are associated to child development and well-being (Broderick and Blewitt, 2003). Baumrind's (1966) theory of parenting style was focused on the control parents executed over their children or *parental demandingness* and on the level parents respond to the child's needs or *parental responsiveness*. She presented three different parenting styles through the combination of these two factors. The different parenting styles are: Authoritative (moderate demandingness and moderate responsiveness), permissive (low demandingness and high responsiveness), and authoritarian (high demandingness and low responsiveness). Later, Maccoby and Martin (1983) added a fourth parenting style known as negligent parenting, characterized by lack or responsiveness and demandingness (Richaud et al., 2013).

Several studies have been made to analyze the role of parenting in predicting academic outcomes in children and adolescents. In general, authoritative parenting characterized as high on demandingness and high on responsiveness would be deemed to facilitate academic outcomes in youth. On the contrary, authoritarian parents characterized by high demandingness in relative absence of responsiveness would provide little support and little motivation for their children to engage academically (Chen et al., 1997; Steinberg et al., 2006). However, few studies have stated that when certain individual factors are controlled, parenting styles would be related to academic performance (Pittman and Chase-Lansdale, 2001). Authors as Joshi et al. (2003) have shown that there exists no significant relationship between parenting styles and academic performance of adolescents. Other authors like Steinberg et al. (1989) and Masud et al. (2016) haven't found direct effects of parenting styles on academic performance but have found that they benefit or work against the latter one, through the influence of certain mediator variables, such as the kind of relationships the adolescents have established with their peers.

While most studies have analyzed the relationship between authoritative and authoritarian parenting styles and academic performance, only a few have attempted to establish specific relationships between the permissive or indulgent style and the functionality of adolescent development. Regarding this, Lamborn et al. (1991) state that adolescents in permissive homes have a high sense of self-confidence but at the same time they present higher substance abuse and greater school misbehavior and are less committed to school. They also establish the need to differentiate between permissive and negligent parents as proposed by Maccoby and Martin (1983). For its part Steinberg et al. (2006) and Mesurado and Richaud (2011) state that adolescents who describe their parents as negligent are less mature, less competent and more troubled than those who describe their parents as authoritative, at the same time those who come from authoritarian homes consistently have a better performance than those who come from indulgent homes. This pattern, they say, remains the same through ethnicity and gender. Due to the scarcity of research that go beyond mentioning the permissive parenting style to study its consequences on the relationships of the adolescent with their peers and on academic behavior and performance, we have decided to study this particular parenting style.

There is scarce research on the process that may explain the links between parenting styles with academic outcomes. We then examine two potential mediator mechanisms, the kind of peer relationships academic and academic self-efficacy, in these relationships.

Parenting styles have also been linked to youth peer relationships (Ladd and Pettit, 2002; Richaud et al., 2011). In general, it was found that authoritarian parenting was positively related with aggression and negatively with peer acceptance and sociability-competence, while authoritative parenting style was positively associated with social adjustment. At the same time, parenting styles of fathers and mothers would predict social adjustment differently. While maternal acceptance was associated with emotional adjustment, paternal acceptance predicted late social and school performance. It was also found that paternal, but not maternal, indulgence significantly predicted difficulties in the social adjustment of children (Chen et al., 2000). This is why we will study separately the relationship between the styles of the father and the mother with the kinds of bond established with the peers.

There is evidence that indicates that poor quality of parenting such as harshness, low warmth, and inadequate monitoring improves the likelihood of having uncooperative and antisocial children (Zhan-Waxler et al., 2008). This increased propensity to associate with other problematic peers have an influence on adolescent behavior problems at school and on academic performance (Santor et al., 2000; Dumka et al., 2009).

Direct bullying implies physical and verbal aggression, while indirect bullying entails relational aggression (Shetgiri, 2013). Relational aggression denotes behaviors aimed at hurting others through the manipulation of relationships, social status, and feelings of belonging or acceptance (Crick and Grotpeter, 1995). For its part, relational victimization is a sub-type of peer victimization that implies being the goal of peer relational aggression, a type of behavior aimed at hurting others through intentional damage or manipulating their interpersonal relationships or by threatening to destroy these relations (Crick et al., 2001). There is a subgroup of victimized children who are oppositional and aggressive (Xu et al., 2003) and who are frequently bullied because their aggressive behavior irritates their peers. Different studies state that relational and physical aggression predicted peer rejection (e.g., Crick et al., 2006; Schwartz et al., 2010; Tseng et al., 2013) and that peer rejection was related to relational and physical victimization (e.g., Crick et al., 1999). Relationally aggressive behaviors tend to increase during the early to middle years of high school (Underwood et al., 2009). Parental monitoring emerged as a protective factor in reducing both victimization and relational aggression (Leadbeater et al., 2008). Instead, negative parenting behavior including abuse and neglect and maladaptive parenting was more likely to associate with children who were victims or with those who at the same time were bully and victims (bully/victim). Finally, positive parenting behavior characterized by good communication, warm relationship, parental involvement and support, and parental supervision were protective against peer victimization (Lereya et al., 2013). Also children who perceive parental support, acceptance, or dedication are less likely to be involved in bullying (Baldry and Farrington, 2005; Ok and Aslan, 2010), while those that perceive little support or that their parents are authoritarian and punitive are prone to develop aggressive behaviors (Baldry and Farrington, 2000; Kawabata et al., 2011). Similarly, the quality of communication between father and child is strongly related to the problems of victimization and violent school behavior, being adolescents that perceive negative communication with their father prone to get involved in school violence (Estévez et al., 2005, 2007).

Based on prior theory that identifies different forms of relationship with peers (e.g., Wang et al., 2015), we examine the links between parenting styles with positive bonds, victimization, and aggressiveness to peers in adolescents.

At the same time, peer relationships was a predictor variable on Hispanic students' academic self-concept (Calero et al., 2014). Furthermore, other researchers have found that peer victimization and perceived academic competence were negatively associated (Thijs and Verkuyten, 2008) whereas positive peer relationships were related with academic domains of self-concept (Marsh et al., 2004, 2011). On the other hand, the kind of relationship with peers also appears to be related to academic self-efficacy. According to Kokkinos and Kipritsi (2012) bullying was negatively correlated with overall self-efficacy and its academic component, while victimization was negatively correlated with overall self-efficacy in children. Also, recent studies (e.g., Andreou and Metallidou, 2004) have shown that bullying and victimization problems were associated with low academic self-efficacy. There is a growing body of research that generally demonstrates positive association between positive peer bonds and academic results. On the contrary, peer victimization was negatively related with academic achievement peer through school engagement (Iyer et al., 2010; Nakamoto and Schwartz, 2010).

According to Bandura's social cognitive theory (Bandura, 1997), self-efficacy is “an individual's convictions about his or her abilities to mobilize the motivation, cognitive resources, and courses of action needed to successfully execute a specific task within a given context” (Stajkovic and Luthans, 1998, p. 66). It exists consistent evidence for the links between academic self-efficacy and academic achievement (e.g., Bandura et al., 2001; Schunk and Pajares, 2009; Galleguillos and Olmedo, 2017). Following this line, Bassi et al. (2007) found that students who scored high on self-efficacy reported higher academic aspirations, spent more time in homework, and primarily associated learning activities with optimal experience as compared to students who scored low on self-efficacy. For their part, Salanova et al. (2005) showed that beliefs of academic self-efficacy are related to high levels of academic engagement. However, the studies that analyze the relationships between parenting styles and the quality of the relationships of adolescents with their peers with academic self-efficacy are scarce. Steinberg et al. (1989) state that adolescents who perceive their parents as warm, democratic, and firm are prone to develop positive attitudes toward, and beliefs about, their achievement, and therefore, they are more likely to do better in school. According to Masud et al. (2016), self-efficacy mediates the relationship of authoritative parenting style and academic performance. Students from authoritative families have higher and significant self-efficacy beliefs as compared to those of authoritarian and permissive families (Strage and Brandt, 1999; Chandler, 2006; Kek et al., 2007; Turner et al., 2009). Furthermore, children from authoritative families have high self-efficacy beliefs, and when they face challenges regarding academic tasks, they handle it effectively (Baumrind and Black, 1967; Baumrind, 1973).

From these theoretical and empirical premises on parenting styles, peer relationships, academic self-efficacy and performance, this study aims to provide insight into a model, based on the hypothesis that authoritative and permissive parenting styles, separately for mother's and father's parenting style, together with kind of peer relationships, may foster or thwart academic self-efficacy and that this in term mediates their relationships with academic performance in late adolescence. Specifically, it contributes to the literature in at least three ways. First, we provide an integrated model of the relationships between parenting styles, different kinds of relationships adolescents-peers, academic self-efficacy, and academic performance. Second, we analyze the mediator role of academic self-efficacy and kind of peer relationships (attachment, aggression, and victimization) in the relationship between authoritative and permissive parenting styles with academic performance. Such analyzes will contribute to highlight the importance of parenting styles in the explanation of academic performance that focuses on the mediator role of peer relationships and academic self-efficacy. Third, we analyze the specific role of permissive parenting style in the development of kind of peer relationships and academic self-efficacy. This analysis will contribute to clarify the importance of the permissive parenting style that is generally named in the literature but not analyzed in its specific effects on peer relationships and academic self-efficacy.

Building on previous research we expected the following hypothesis:

1. The first hypothesis is that the authoritative parenting style (Wave 1, W1) is positively related to positive relationships with adolescent peers (W2) (Richaud et al., 2011), whereas the permissive style (W1) is negatively related to positive adolescent peer relationships and positively related with victimization and aggression/bullying (W2) (Chen et al., 2000).
2. The second hypothesis postulates that the authoritative parenting style is positively related to academic self-efficacy (Masud et al., 2016), whereas the permissive style is negatively related to academic self-efficacy (Strage and Brandt, 1999; Chandler, 2006; Kek et al., 2007; Turner et al., 2009).
3. The third hypothesis postulates that adolescent positive peer relationships (W2) are positively related to academic self-efficacy (W3) (Marsh et al., 2011) whereas victimization and aggression (W2) are negatively related to academic self-efficacy (W3) (Thijs and Verkuyten, 2008).
4. The fourth hypothesis assumes that academic self-efficacy is positively related to academic performance (Schunk and Pajares, 2009; Galleguillos and Olmedo, 2017).
5. The fifth hypothesis postulates that parenting styles are related to academic performance through peer relations and academic self-efficacy. Peer relationships (attachment, aggression, and victimization) (W2) and academic self-efficacy (W3) mediate the relationship between parenting style and academic performance (W3) (Ladd and Pettit, 2002; Marsh et al., 2004, 2011; Schunk and Pajares, 2009; Richaud et al., 2011; Galleguillos and Olmedo, 2017). The hypothesis assumes differences for fathers and mothers (Chen et al., 2000).

## Materials and methods

### Participants

Five hundred Spanish adolescents were evaluated in a three-wave longitudinal study in Valencia, Spain. However, 400 and 17 adolescents fully completed all three surveys. The final sample consisted of 192 boys and 225 girls. In the first wave, adolescents were either in the third year of secondary school (81 boys and 85 girls) or the fourth year of secondary school (111 boys and 140 girls). The mean age in the first wave was 14.70 (*SD* = 0.68; range = 13–16 years). This study monitored participating adolescents in three waves: Wave1 (W1), when adolescents were in 3rd, or 4th grade; Wave 2 (W2), when adolescents were in 4th or 5th grade; and Wave 3 (W3), when adolescents were in 5th or 6th grade. Participating schools were randomly selected from the list of all schools in Valencia with students enrolled in compulsory secondary education. In total, 11 schools participated in the study.

The majority of participants came from two-parent households where parents were married (83.7% married; 13.2% divorced). Regarding the educational level, 21.8% of mothers had less than a secondary school diploma, 42.2% had a secondary school diploma or equivalent and 30.7% had some university education. Likewise, 24% of fathers had less than a high school diploma, 41% had a high school diploma or equivalent, and 28.7% had some university education. 86.6% of the students self-identified themselves as being from Spain. Small percentages of the adolescents self-identified themselves as being from Latin America (e.g., 3.4% from Ecuador, 2% from Colombia, and 1.1% from Bolivia) and Eastern European countries (e.g., 1.7% from Romania).

### Procedure

Approval from the School Council and written informed consent from parents were obtained. The research followed all ethical guidelines, respecting respondents' anonymity for both data collection and data analysis. Participation by students was voluntary and they were free to decline to participate in the study. The instruments were administered by trained researchers in the classroom in 50-min sessions during school hours. The annual evaluations took place in three consecutive years during the first trimester of the school year.

### Measures

Consistent with prior approaches to operationalizing responsive and demanding parenting dimensions (Simons and Conger, 2007), adolescents' third grade reports of parenting were previously used to identify parenting styles based on the dimensions of parental responsiveness (i.e., high acceptance and low harshness) and demandingness (i.e., high consistent discipline, and monitoring).

To define authoritative style we used adolescents' reports of mothers' and fathers' acceptance and moderated monitoring, and to define permissive style we used adolescents' reports of mothers' and fathers' acceptance and low control or extreme autonomy. Acceptance, moderated monitoring, and extreme autonomy were assessed using the Child Report of Parental Behavior Inventory (Schaefer, 1965; Samper et al., 2006; CRPBI). This instrument evaluates the child's perceptions of family discipline in relationships with the child's mother and father. Example items are, “He (she) controls if I have tidied my room,” “He (she) let's me arrive at any time” and “He (she) doesn't ask where I go or with whom.” Participants indicated their agreement with each statement using a three-point scale (*completely agree, sometimes, completely disagree*). Students responded once thinking of their father and once thinking of their mother. For this study, we selected two factors from the instrument. The first factor was *support, communication, and moderated monitoring (authoritative style)*, which describes relationships based on feelings of emotional support from the father and mother, the sending of messages of affect and support, encouragement of autonomy based on discipline, and good communication between parents and children. The second factor was *extreme autonomy (permissive style)*, which describes relationships based on extreme laisser-faire, complete freedom without rules or limits. The scales had acceptable indices of reliability for all three evaluations (W1, W2, and W3, respectively—*support, communication, and moderate control* mother: alpha = 0.88; 0.90; 0.91 and father alpha = 0.89; 0.90; 0.92; *extreme autonomy* mother alpha = 0.80; 0.76; 0.79 and father alpha = 0.78; 0.80; 0.78).

Peer Attachment (from the *Inventory of Parent and Peer Attachment* by Armsden and Greenberg, 1987). This instrument evaluates behavioral and affective/cognitive dimensions related to peer attachment. Example items are, “My friends respect my feelings,” “I tell my friends about my problems and issues,” “If my friends know that something is worrying me, they ask me about it.” Cronbach's alpha for this instrument was 0.75 at W1, 0.83 at W2, and 0.84 at W3.

Victimization (from the *Kit at School*, Buhs et al., 2010). In this study, six items have been used to collect the three victimization factors described in the Buhs et al. (2010) scale that refer to *relational victimization* (refers to behaviors that seek to harm through “intentional manipulation and damage to the relationship between peers”), *manifests* (includes physical (e.g., beating) and verbal (e.g., insults) behaviors aimed at directly damaging others; and *social exclusion*. Students have to answer in a Likert scale of five alternatives (1 = “almost never,” 3 = sometimes and 5 = “almost always”). Examples of items are: *How often do peers in your school: “make fun of you or insult you* (manifest)”; *tell lies, gossip, rumours or spread bad news about you?*” (Relational), “*do they leave you out of conversations, games or activities?* (Social exclusion).” Cronbach's alpha for this study was 0.70 at W1, 0.78 at W2, and 0.81 at W3.

Physical and Verbal Aggression Scale (Caprara and Pastorelli, 1993; Del Barrio et al., 2001). This instrument uses 20 items to assess behaviors that harm others physically or verbally. Respondents indicate the frequency with which the behavior in each statement occurs (*often, sometimes, never*). Example items are, “I hit, kick and punch” and “I threaten others.” Cronbach's alpha for this research was 0.81 at W1, 0.82 at W2, and 0.83 at W3).

Academic Self-efficacy (ad hoc questionnaire). This instrument uses six items to evaluate the self-perception of three factors related to academic performance: effort, motivation, and performance. The students have to rate on a scale of 1–10. Example items are, “How do you consider your academic performance?” and “do you consider yourself a good student?.” Cronbach's alpha for this study was 0.75 at W1, 0.78 at W2, and 0.78 at W3.

Academic Performance (*ad hoc* questionnaire). Through an instrument built ad hoc to record the assessment of the teacher or tutor on the effort, motivation and performance of each of the students. The teacher/tutor have to rate on a scale of 1 to 10 their perception of each student regarding three factors: effort, motivation and performance. Example items are, “How do you consider his/her academic performance?” and “do you consider him/her a good student?.” Cronbach's alpha for this study was 0.91 at W1, 0.91 at W2, and 0.92 at W3.

### Analysis plan

First of all, SPSS 22 was used to analyse means and standard deviations. Pearson correlation analysis was carried out to test the relationships among variables. Finally, structural equations modeling (SEM) in AMOS 17.0 (SPSS Inc., 2007) was employed to explore the proposed models, using maximum likelihood method. The following goodness-of-fit indices were used: chi-square, chi-square divided by degrees of freedom (χ^2^/df), goodness-of-fit index (GFI), and Bentler comparative fit index (CFI). Root mean residual (RMR) and root mean square error of approximation (RMSEA) were used to measure error. We analyzed two mediators (peer relations: attachment, victimization or aggression, and academic self-efficacy) between two independent variables (parent authoritative and parent permissive) and one dependent variable (academic performance).

## Results

### Descriptive statistics and correlations

There were no significant differences in parenting style, peer relationships, and academic self-efficacy and academic performance between adolescents with complete data and their counterparts without full data. Descriptive statistics and Pearson correlations among parenting styles, peer relationships, and the academic variables corresponding to W1, W2, and W3 are presented in Table 1.

**Table 1:** Descriptive statistics and Pearson correlations.

	**1**	**2**	**3**	**4**	**5**	**6**	**7**	**8**	**9**
1. Authoritative Mother W1	–	0.65^**^	0.07	−0.03	0.25^**^	−0.06	−0.09	0.28^**^	−0.05
2. Authoritative Father W1		–	−0.04	0.03	0.18^**^	−0.14^**^	−0.15^**^	0.23^**^	−0.07
3. Permissive Mother W1			–	0.60^**^	0.06	−0.01	0.05	0.02	0.12^*^
4. Permissive Father W1				–	−0.04	−0.05	0.05	0.01	0.04
5. Peer Attachment W2					–	−0.23^**^	−0.20^**^	0.32^**^	0.06
6. Victimization W2						–	0.19^**^	−0.05	−0.02
7. Aggression W2							–	−0.36^**^	0.00
8. Academic Self-efficacy W3								–	0.21^**^
9. Academic Performance W3									–
M (SD)	2.22 (0.39)	2.10 (0.41)	1.61 (0.34)	1.61 (0.37)	3.71 (0.57)	1.87 (0.33)	1.30 (0.24)	45.97 (6.51)	44.54 (8.65)

* *p < 0.05*,

** *p < 0.01; W1 = Wave 1, W2 = Wave 2, W3 = Wave 3*.

### Structural equation model

**Figure 1:**
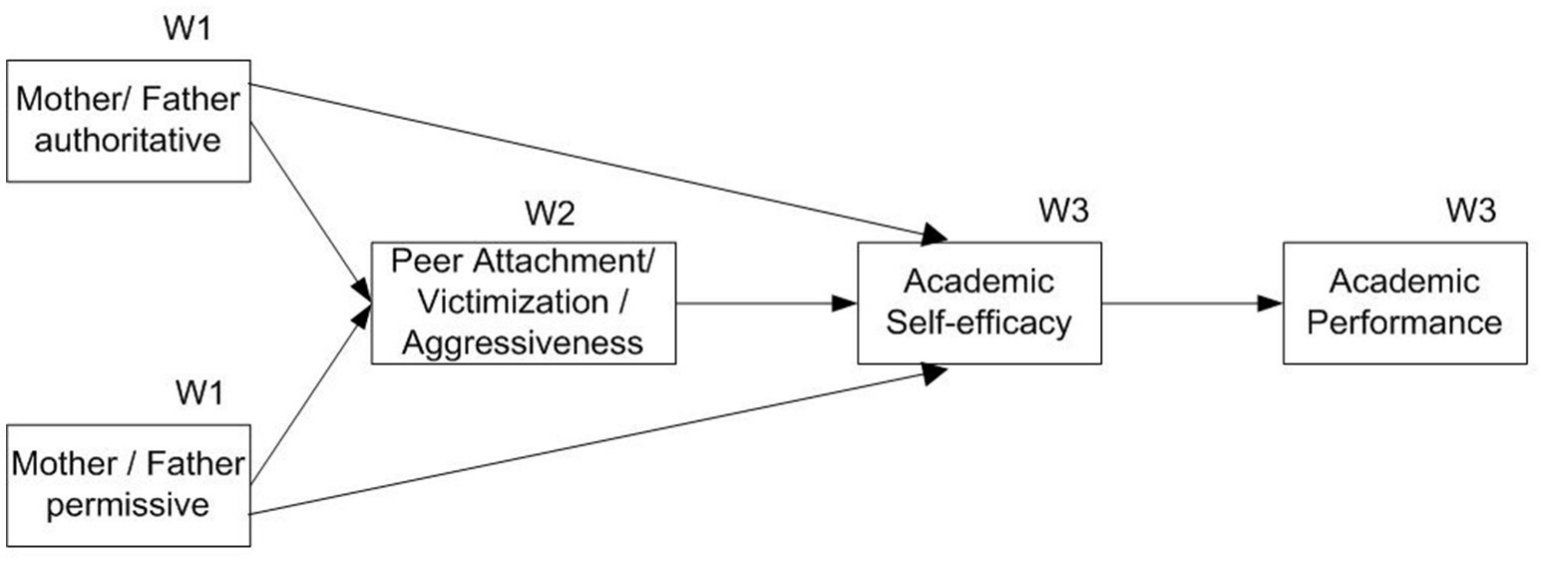
Model graphic corresponding to longitudinal study.

Models were run separately for mother's parenting style and father's parenting style and for adolescents peers attachment, victimization, and aggressiveness (See Figure 1). The models included direct relations among parenting styles W1, kind of peer relationshipsW2, academic self-efficacy W3, and academic performance W3. Direct paths from parenting stylesW1 and academic self-efficacyW3 were also included. Finally the following indirect and total effects were studied.

Total effects:

X1 (parent authoritative) W1–Y (academic performance) W3
X2 (parent permissive) W1–Y (academic performance) W3

Indirect or mediating effects:

X1W1–M1W2 (peer relations)–M2 (academic self-efficacy) W3–YW3
X2W1–M1W2–M2W3–YW3
X1W1–M2W3–YW3
X2W1–M2W3–YW3

In all models, the error variance of exogenous parenting styles were allowed to correlate.

### Main model findings

The different models fit the data well (See Table 2).

**Table 2:** Fit indexes corresponding to the models of longitudinal study.

**Models**	**χ^2^**	***p***	***df***	**χ^2^/*df***	**GFI**	**CFI**	**RMR**	**RMSEA**
1	6.35	0.10	3	2.12	0.99	0.98	0.09	0.05
2	6.78	0.08	3		0.99	0.96	0.08	0.05
3	6.44	0.09	3	2.15	0.99	0.96	0.09	0.05
4	7.07	0.07	3		0.99	0.91	0.09	0.05
5	8.81	0.03	3	2.94	0.99	0.96	0.09	0.07
6	8.682	0.03	3		0.99	0.94	0.09	0.06

*Model 1 Authoritative and permissive mother W1—peer attachment W2; Model 2 Authoritative and permissive father W1—peer attachment W2; Model 3 Authoritative and permissive mother W1—victimization W2; Model 4 Authoritative and permissive father W1—victimization W2; Model 5 Authoritative and permissive mother W1—aggressiveness W2; Model 6 Authoritative and permissive father W1—aggressiveness W2*.

In the models examining mother's parenting styles W1 results indicated that adolescents with authoritative mother and father W1 are more likely to develop attachment with peers W2 but only father authoritative style is negatively associated to adolescent victimization and aggressiveness. On the other hand, the mother permissive style W1 negatively relates to academic self-efficacy in W3, but only in the model of mother W1—victimization W2. Furthermore, permissive mother W1 was a significant positively predictor of adolescent aggressiveness W2, and a significant negative predictor of adolescent peer attachment W2 but it is not related to adolescent victimization W2.

In both mother and father parenting style models, adolescent aggressiveness W2 is associated to lower academic self-efficacy W3 and the adolescent attachment W2 is associated to greater academic self-efficacy W3. On the other hand, adolescent victimization W2 does not relate to academic self-efficacy W3. In addition, adolescent academic self-efficacy W3 is related to academic performance in both models (See Table 3).

**Table 3:** Standardized coefficients of paths corresponding to the models of longitudinal study.

	**Peer attachment W2**	**Academic Self-efficacy W3**	**Academic performance W3**
Authoritative Mother W1	0.21^***^	0.19^***^	
Permissive Mother W1	−0.14^**^	−0.09	
Peer attachment W2		0.26^***^	
Academic self-efficacy W3			0.21^***^
Authoritative Father W1	0.19^***^	0.17^***^	
Permissive Father W1	−0.04	0.01	
Peer attachment W2		0.29^***^	
Academic self-efficacy W3			0.21^***^
	Victimization W2		
Authoritative Mother W1	−0.03	0.24^***^	
Permissive Mother W1	0.10	−0.12^**^	
Victimization W2		−0.02	
Academic self-efficacy W3			0.21^***^
Authoritative Father W1	−0.14^**^	0.22^***^	
Permissive Father W1	0.05	0.00	
Victimization W2		−0.02	
Academic self-efficacy W3			0.21^***^
	Aggressiveness W2		
Authoritative Mother W1	−0.04	0.23^***^	
Permissive Mother W1	0.21^***^	−0.07	
Aggressiveness W2		−0.32^***^	
Academic self-efficacy W3			0.21^***^
Authoritative Father W1	−0.15^**^	0.18^***^	
Permissive Father W1	0.05	0.02	
Aggressiveness W2		−0.33^***^	
Academic self-efficacy W3			0.21^***^

** *p < 0.01*,

*** *p < 0.001; W1, Wave 1; W2, Wave 2; W3, Wave 3*.

Bootstrap 90% confidence intervals (CIs) were used to test indirect effects (MacKinnon et al., 2002). In Table 4 appear indirect effects values, significant level, and CI lower and upper bounds (indicating that 90% of the population lies between lower bound and upper bound with 5% less than lower bound value and 5% greater than upper bound value). The indirect relations between parenting styles, academic self-efficacy, and academic performance, mother authoritative via academic self-efficacy indirect effect = 0.13, CIs [0.09, 0.17], *p* < 0.01, and father authoritative via academic self-efficacy indirect effect = 0.05, CIs [0.02, 0.08], *p* < 0.01 were significant. The total effects authoritative mother—academic performance and authoritative father —academic performance were significant, authoritative mother total effect = 0.06, CIs [0.03, 0.10], *p* < 0.01 and authoritative father total effect = 0.05, CIs [0.02, 0.08], *p* < 0.01.

**Table 4:** Standardized indirect effects.

		**CI Lower bound**	**CI Upper bound**
Authoritative mother—academic performance/peer attachment/academic self-efficacy	0.017^**^	0.007	0.031
Permissive mother—academic performance/peer attachment/academic self-efficacy	0.003	−0.003	0.010
Authoritative father—academic performance/peer attachment/academic self-efficacy	0.013^**^	0.005	0.023
Permissive father—academic performance/peer attachment/academic self-efficacy	−0.003	−0.009	0.003
Authoritative mother—academic performance/victimization/academic self-efficacy	0.001	−0.001	0.003
Permissive mother—academic performance/victimization/academic self-efficacy	0.000	−0.001	0.001
Authoritative father—academic performance/victimization/academic self-efficacy	0.048^**^	0.023	0.079
Permissive father—academic performance/victimization/academic self-efficacy	0.000	−0.014	0.021
Authoritative mother—academic performance/aggression/academic self-efficacy	0.007	0.000	0.016
Permissive mother—academic performance/aggression/academic self-efficacy	−0.004	−0.001	0.002
Authoritative father—academic performance/aggression/academic self-efficacy	0.011^**^	0.004	0.020
Permissive father—academic performance/aggression/academic self-efficacy	−0.004	−0.010	0.001

** *p < 0.01; 90% Confidence Interval (CI)*.

## Discussion

Due to the scarcity of research about the process that may explain the links between parenting style and academic performance we have hypothesized that this relation will appear through the mediator effect of peer relations and academic self-efficacy. At the same time, we were interested in the relationship between permissive parenting style and the kind of relationships developed with peers in adolescence, in deepening the study of this relationship. We have also assumed that there would be a relationship between the parenting style and the kind of relationship between the adolescent and their peers with academic self-efficacy, since the main development contexts of self-efficacy in individuals are family, peers, and school. The interactions that arise in such contexts nourish in a significant way the resources that appear in the life of the individual, contributing to the development of an adequate or inadequate sense of self-efficacy and allowing the evolution from the extreme control to personal self-regulation (Pastorelli et al., 2001). However, research focusing on the family history of the development of self-efficacy beliefs is scarce (Schneewind, 1999; Caprara et al., 2005). On the other hand there are few studies about the relationships between parenting styles and kind of adolescent peer relationships with academic self-efficacy.

The model was analyzed in three waves that range from early-mid adolescence to late adolescence to determine if the pattern of relationships studied in early-mid adolescence stays the same or suffers differences and what are those up to the late adolescence. The parenting style perceived in the first wave is analyzed, as well as the kind of relationship of the adolescent with their peers in the second wave and the academic self-efficacy and performance in the third wave, to observe if the bonds with significant others, parents and peers, maintain their influence on adolescent functioning until late adolescence.

The results have shown that, as the first hypothesis proposes, the authoritative parenting style is positively related to positive relationships or attachment with adolescent peers. On the contrary, the permissive parenting style is not significantly related with peer relations. The results obtained partially support the first hypothesis as they indicate that despite the authoritative style of both the father and the mother are important positive predictors of the attachment of the adolescent to their peers, only the authoritative style of the father prevents the victimization of the adolescent and aggressiveness (Leadbeater et al., 2008; Lereya et al., 2013), meaning that the support and the affection of the father appear with a higher frequency opposed to dysfunctional bonds. This would be due to the expression of warmth, affection and concern to cover the needs of the children, while exercising a moderate control which is accepted by the children as an expression of care (Richaud de Minzi, 2007), generating a positive model of attachment that would be carried out in the various social relationships and in particular with peers. At the same time, our results show that this kind of parental bond prevents the adolescent from developing poor relationships with peers like aggressiveness or victimization (Leadbeater et al., 2008), which also appear in this study significantly related, agreeing with what stated by Xu et al. (2003) and Zhan-Waxler et al. (2008).

Regarding authoritative and permissive parenting relationships with academic self-efficacy, the results partially support the second hypothesis. A direct effect of the authoritative style of the mother and the father over the development of academic self-efficacy has been found. These findings coincide with those of Caprara et al. (2005) who suggest that as long as adolescents feel that they are satisfactorily interacting with their parents, they are more likely to trust them about their concerns, activities and the dilemmas they face in their social experiences away from home. From this perspective, it is expected that the parenting styles play a fundamental role in fostering an open communication between adolescents and their parents, in preventing conflicts from escalating, in promoting adequate monitoring, in promoting self-regulatory models in different aspects, among them the academic one, and finally in leading the adolescents toward a satisfactory adult life. On the contrary, neither the father's nor the mother's permissiveness are related to academic self-efficacy, which may be due to the lack of monitoring, which does not generate self-regulation and self-confidence, but does not prevent it as an excessive control in the authoritarian parental style (Chandler, 2006; Kek et al., 2007; Turner et al., 2009). It simply does not relate to the development of a belief in positive control of the situation.

The results partially support the third hypothesis that postulates that adolescent attachment with peers is positively related to academic self-efficacy whereas victimization and aggression are negatively related to academic self-efficacy. We have found direct relations between the quality of the bond with peers (attachment and aggression) and the academic self-efficacy except in the case of the victimization which has no negative influence on academic self-efficacy. These results coincide with those of different authors (Andreou and Metallidou, 2004; Marsh et al., 2011; Kokkinos and Kipritsi, 2012). In regards to aggressiveness, adolescents with a high aggressive behavior present low academic self-efficacy and an elevated negative perception from their peers, finding positive correlations between high antisocial self-assessed behaviors and academic problems (Garaigordobil, 2005). In addition, in regards to attachment, positive peer relationships were associated with academic self-efficacy (Marsh et al., 2004, 2011).

The results support, in agreement with the literature (Bandura et al., 2001; Bassi et al., 2007; Schunk and Pajares, 2009) the fourth hypothesis that assumes that academic self-efficacy is positively related to academic performance. Finally, in general we have not found direct relations between the parenting styles or the kind of relationship with peers with academic performance, but that they are mediated by academic self-efficacy as stated by Masud et al. (2016). Even though we have not included the study of the direct relation between parenting styles and kind of relation with peers with academic performance in the models we have presented, in previous studies we found that there was no significant relation, and for this reason we excluded these effects to simplify the models.

The results partially support the fifth hypothesis that postulates that parent styles (W1) are related to academic performance (W3) through peer relations and academic self-efficacy. Peer relationships (attachment, aggression, and victimization) (W2) and academic self-efficacy (W3) mediate the relationship between parenting style and academic performance (W3). In addition, the hypothesis assumes differences for fathers and mothers. The results show that peer attachment and academic self-efficacy are mediator variables between authoritative parenting style, from both parents, and academic performance. However, when victimization and aggression are the mediator variables, there are differences between fathers and mothers. It would seem that to the extent that the mother is not significantly related to victimization or aggression, they cancel out the effect of the mother's authoritative style on academic performance. In contrast, in relation to the father, having a negative relationship with both victimization and aggression, maintains a positive and significant effect on the child's academic performance. On the other hand, indirect effects of authoritative parenting style over academic performance, both via peer relations and academic self-efficacy and via self-efficacy alone were significant, but not in the case of permissive style. The same happened when analyzed the total effects of parenting styles on academic performance. Only the total effect was significant in the case of authoritative parenting style. These results support previous results that explain that authoritative parenting style generates a positive model of attachment in their children (Richaud de Minzi, 2007; Leadbeater et al., 2008), that may also explain the higher and significant self-efficacy beliefs of students from authoritative families as compared to permissive families (Baumrind and Black, 1967; Baumrind, 1973; Strage and Brandt, 1999; Chandler, 2006; Kek et al., 2007; Turner et al., 2009).

Despite indirect effects were not found for the permissive parenting styles from both parents, we have found that only the mother's is a positive predictor of aggression toward peers and a negative predictor of and attachment relationship with peers. At the same time, these peer relations are predictors of academic self-efficacy. Given that the permissive style is characterized by lack of control which, even accompanied by affection, can be perceived as unconcerned to respond to the needs of the child, it would generate an insecure bond that would be expressed as a poor internal control and a more impulsive behavior. According to Schneewind (1999), parents who tend to have children with a greater internal control orientation are those who offer a stimulating family environment, who respond consistently and appropriately to their children's behavior, as well as those who promote independence and autonomy, use more inductive discipline techniques, and relate emotionally in a comforting manner. On the contrary, parental permissiveness would relate to intrusive, often aggressive interactions of adolescents, as if that unconcernedness perceived in the mother by satisfying adolescent needs or setting limits that signify care, would later generate in adolescence an externalization of feelings of anger rather than its internalization in the form of affective inhibition and depression. It is possible that, as due to cultural beliefs and practices, a higher protection and response to the needs of the child it is expected from the mother than from the father, the lack of these characteristics in the bond with the mother has more consequences on the functioning of the adolescent than their lack in the bond with the father.

To sum up, parenting styles relate to the kind of relationships the adolescents have with their peers and with academic self-efficacy, especially, authoritative parenting style from both parents relates to the peer attachment and academic self-efficacy, and father's authoritative style relates to aggression and victimization. In addition, academic self-efficacy is a mediator between authoritative parenting style and academic performance. Finally, the mother's permissive style is an important positive predictor of aggression of adolescents and a negative predictor of attachment of the adolescent to their peers.

### Strengths, limitations, and future directions

This article presents an integration model of the bonds with the significant others, parents and peers, academic self-efficacy and performance in early-mid adolescence until late adolescence. We have also analyzed the way in which the authoritative and permissive parenting styles of both parents separately affect the development of attachment with peers or conversely their aggressiveness or victimization. The permissive style has been particularly studied which in the mother shows to have very negative consequences for the development of the relationships of the adolescent with their peers, having a predictor role of aggressiveness and victimization of the adolescent. The study of permissive parenting style becomes important in the context of a society in which, whether due to cultural changes in the concept of relationship parent-child or due to conflict between the parents which lead to competition over the love of the child, the limits and control needed by the child to develop an internal control, self-confidence, and self-efficacy beliefs are left behind. The hypothesized mediator role of quality of the bond with peers and academic self-efficacy between parenting styles and academic performance has also been studied.

A limitation of the present study is that only encompasses mainly the end of early adolescence, mid adolescence and the beginning of late adolescence. Consequently it would be necessary in future research to analyze the relationships studied here during a period ranging from pre-adolescence to the end of late adolescence. On the other hand, the present research was carried out in a specific culture. Collecting data from more diverse samples including different cultural contexts should be considered in the future. Furthermore, the use of self-reports instruments to define the kind of parenting style was used adolescents' reports of mothers' and fathers' behavior is another limitation.

Even though we have studied the parenting styles of the mother and the father separately, it would be important to also analyze their relations to male and female adolescents, as the cultural rearing patterns are different. Likewise, to study the relationships between the adolescent and their peers taking gender into consideration is an important consideration when studying those social relationships.

## Ethics statement

The participation of the adolescents was voluntary and anonymous, taking into consideration all ethical principles pertaining to research with human beings included in the Helsinki Declaration, under the current regulations. The research project had a favorable response from the university ethics committee because it is required for the concession of these studies (GVPROMETEO/2015/003, PSI2016-78242-R and AICO/2016/090).

## Author contributions

AL and EM: Materials and Method and Results. MR: Introduction and Discussion.

### Conflict of interest statement

The authors declare that the research was conducted in the absence of any commercial or financial relationships that could be construed as a potential conflict of interest.
